# Dermatology

**Published:** 1879-05

**Authors:** 


### DERMATOLOGY.

CHRYSOPHANIC ACID IN PSORIASIS. Neumann.—(*L' Union Médicale*, Feb 8, 1879.)

| | |
|---|---|
| Chrysophanic acid | 6 |
| Prepared lard | 30 |

Mix.

The ointment is spread upon lint, or if the skin is infiltrated, on strips of cloth, which are then applied to the diseased patches. After three or four dressings the scales disappear, aud the subjacent skin is perfectly white, but in a few days resumes its natural aspect. When the psoriasis is accompanied by excessive infiltration, ten or twelve applications are necessary. For herpes tonsurans and pityriasis versicolor, three inunctions are generally sufficient; but it is for psoriasis that chrysophanic acid has proved the most efficacious. Unfortunately, the cure is not radical, and the eruption may reappear later. Chrysophanic acid should not be applied to the face except with reserve, on account of the change of color which it may cause in the skin and hair. It has, besides the inconvenience of staining the linen. Ointment of much less strength may be sufficiently active to fulfill all the indications.

